# Brain Delivery of a Potent Opioid Receptor Agonist, Biphalin during Ischemic Stroke: Role of Organic Anion Transporting Polypeptide (OATP)

**DOI:** 10.3390/pharmaceutics11090467

**Published:** 2019-09-10

**Authors:** Thamer H Albekairi, Bhuvaneshwar Vaidya, Ronak Patel, Saeideh Nozohouri, Heidi Villalba, Yong Zhang, Yeon Sun Lee, Abraham Al-Ahmad, Thomas J Abbruscato

**Affiliations:** 1 Department of Pharmaceutical Sciences, Texas Tech University Health Sciences Center School of Pharmacy, Amarillo, TX 79106, USA; 2 Department of Pharmacology and Toxicology, College of Pharmacy, King Saud University, Riyadh 12372, Saudi Arabia; 3 Department of Pharmacology, University of Arizona, College of Medicine, Tucson, AZ 85724, USA

**Keywords:** biphalin, organic anion transporting polypeptide, blood-brain barrier, transport mechanism, ischemic stroke

## Abstract

Transporters (expressed) at the blood-brain barrier (BBB) can play an essential role in the treatment of brain injury by transporting neuroprotective substance to the central nervous system. The goal of this study was to understand the role of organic anion transporting polypeptide (OATP1; OATP1A2 in humans and oatp1a4 in rodents) in the transport of a potent opioid receptor agonist, biphalin, across the BBB during ischemic stroke. Brain microvascular endothelial cells (BMECs) that were differentiated from human induced pluripotent stem cells (iPSCs) were used in the present study. The effect of oxygen-glucose deprivation (OGD) and reperfusion on the OATP1 expression, uptake, and transport of biphalin was measured in induced pluripotent stem cells differentiated brain microvascular endothelial cells (iPSC–BMECs) in the presence and absence of an OATP1 substrate, estrone-3-sulfate (E3S). Biphalin brain permeability was quantified while using a highly sensitive liquid chromatography-tandem mass spectrometry (LC-MS/MS) method. It was found that iPSC-BMECs expressed OATP1. In vitro studies showed that biphalin BBB uptake and transport decreased in the presence of an OATP1 specific substrate. It was also observed that OGD and reperfusion modulate the expression and function of OATP1 in BMECs. This study strongly demonstrates that OATP1 contributes to the transport of biphalin across the BBB and increased expression of OATP1 during OGD-reperfusion could provide a novel target for improving ischemic brain drug delivery of biphalin or other potential neurotherapeutics that have affinity to this BBB transporter.

## 1. Introduction

Stroke or brain ischemia consists of a pathophysiologic state where blood supply to the brain is temporarily or permanently obstructed. It remains the fifth leading cause of death in the United States and the third leading cause of disability worldwide [1]. The identification of novel neuroprotective therapies to be used both during stroke and after blood clot reduction, either by thrombolysis therapy or mechanical thrombectomy, are a major therapeutic opportunity to facilitate recovery from a brain vascular injury. The primary drug target for any potential neuroprotective agents is the area directly around infarct region, called penumbra. It is crucial to transport these agents across the blood-brain barrier (BBB) to reach their respective targets to ensure the neuroprotective activity during ischemia-reperfusion injury. Although both small and large molecules have been being investigated as neuroprotective agents, only small molecules that are lipid soluble and have a molecular weight of less than 400 Da can cross the BBB, whereas large molecules cannot penetrate brain endothelial cells [2]. This transport restriction by the BBB stops 95% of investigational molecules for drug development. However, endogenous transporters can participate in the brain movement of hydrophilic and high molecular weight agents. Hence, it is critical to understand and study the localization and functional expression of endogenous BBB transporters and how disease alters the expression. This could help to maximize neuroprotective drug delivery and optimize the dose to be transported during stroke-reperfusion to ensure that brain protection is given at the optimum time [3].

Opioid receptors (ORs) (mu OR (MOR), delta OR (DOR), and kappa OR (KOR)) have gained extensive interest of researchers for their role as neuroprotective targets and have been reported to demonstrate potential neuroprotective activity in ischemic stroke by several overlapping mechanisms [4]. Our lab has communicated several studies that reported the neuroprotective potential of biphalin, an ultra-potent, non-selective, opioid agonist, in models of both permanent and transient ischemic stroke [5,6,7,8]. The neuroprotective efficacy of biphalin was observed to be better than other OR selective agonists. Earlier, it has been reported that biphalin (a hydrophilic molecule with molecular weight of 909 g/mol) crosses the BBB through passive transcellular diffusion and partially via carrier-mediated transport, and 0.05% of injected dose was accumulated in the brain [9]. This study also demonstrated that an undefined saturable mechanism partially contributes to the transport of biphalin across the BBB. However, no study has fully defined this transport mechanism or reported biphalin brain distribution in stroke models, to help explain its excellent therapeutic efficacy in preclinical models of stroke. Several groups have reported that the BBB transporter expression is altered during ischemic reperfusion injury [10,11,12,13,14], and recently reviewed by our group [15]. For this study, we planned to decipher the mechanism of biphalin transport across the BBB during ischemic stroke.

Structurally, biphalin is a peptide that is composed of bivalent enkephalin pharmacophores, each of which is composed of four amino acids. The amino acids are Tyrosine, D-Alanine, Glycine, and Phenylalanine, with the two enkephalin pharmacophores being linked together by a hydrazine bridge [16,17]. The two last amino acid-tails of biphalin are tyrosine; this chemical feature increased our interest in the possibility that the organic anion transporting polypeptide (OATP1) could be involved in the transport of biphalin during ischemic stroke conditions. We hypothesized that OATP1 could contribute in the transport of biphalin across the ischemic BBB. Well known OATP1 substrates, like thyroid hormones (T3 and T4) and other analgesic peptides, deltorphin II, and [D-Pen^2^, D-Pen^5^] encephalin (DPDPE), all have tyrosine residues similar to biphalin, although the exact structural requirements for qualification as a substrate of OATP1 is not known [18,19,20]. OATP1 are sodium-independent transporters, which are governed by the electrochemical gradient or the transmembrane concentration gradient of the substance being transported [21]. Although the mechanism of OATP1 mediated transport remains controversial, a rocker-switch mechanism has been suggested as a common mechanism to transport the substrates through a central, positively charged pore in OATPs [22]. OATP1 mediates the transport of a broad range of substrates, including endogenous compounds and therapeutic molecules, for example, bile salts, hormones, steroid conjugates, peptides, statins, etc. [23].

Recently, it has also been reported by Thompson et al. 2014 that the expression of oatp1a4, which is an ortholog of human OATP1A2 (OATP1) in rat, increased during hypoxia-reoxygenation conditions [11]. It was observed that after 1 h hypoxia coupled to both 10 min. and 1 h reoxygenation the expression of oatp1a4 in rat isolated brain microvessels increased. It has also been observed that brain uptake of oatp substrates, taurocholate, and atorvastatin increased when perfused at 10 min. after 1 h of hypoxia. These studies suggest that increased OATP1 expression could be utilized to enhance the brain delivery of therapeutics during ischemia-reperfusion injury.

In the present study, we sought to determine the effect of an in vitro model of ischemic stroke (oxygen-glucose deprivation, OGD) on the expression profile of OATP1 and its contribution in the transport of biphalin across the BBB during ischemic stroke. This is the first study where human induced pluripotent stem cells differentiated brain microvascular endothelial cells (iPSC-BMECs) were used as an in vitro cell model to determine the effect of stroke conditions on the expression of OATP1 and its involvement in the transport of biphalin across the BBB. Stem cells have been reported to demonstrate better barrier tightness and provide high fidelity expression of BBB related transporters and receptors as compared to immortalized BBB cells models [24,25]. Further, stem cell derived models serve as a continuous source of BBB cells with minimal batch-to-batch variability [26,27,28]. In addition, patient derived stem cells have the ability to allow for phenotypic disease modeling at BBB in which genetic components of the disease can be captured [29]. These in vitro experiments will provide foundational experiments to test and identify the ability of biphalin to utilize a known saturable mechanism to enter the ischemic brain and help to provide an initial time-window of transport.

## 2. Materials and Methods

### 2.1. Cell Culture: iPSCs Differentiation to BMECs

hCMEC/D3 cell line (Millipore Sigma, Burlington, MA, USA) [30], bEnd.3 (ATCC, Manassas, VA, USA) [31] and SHSY5Y cells (ATCC, Manassas, VA, USA) were cultured according to reported protocols [32]. Undifferentiated stem cells (IMR-90 c4) (WiCell, Madison, WI, USA) were maintained on feeder-free condition using matrigel (Corning Inc., Corning, NY, USA) and Essential-8 medium (Thermo Fisher, Waltham, MA, USA). IMR-90 cells were differentiated in to BMECs following an earlier reported protocol [33]. Briefly, undifferentiated stem cells were plated at 20,000 cells/cm^2^ on six well tissue culture treated dishes to start the differentiations. At the end of three days of expansion with Essential-8 medium, endothelial differentiation was initiated while using unconditioned medium [UM: Dulbecco’s modified Eagle’s medium/F12 with 15 mM HEPES (Thermo Fisher, Waltham, MA, USA), 20% knockout serum replacement (Thermo Fisher, Waltham, MA, USA), 1% non-essential amino acids (Thermo Fisher, Waltham, MA, USA), 0.5% Glutamax (Thermo Fisher, Waltham, MA, USA), and 0.1 mM b-mercaptoethanol (Sigma-Aldrich, St. Louis, MO, USA)] for six days. To expand the endothelial cell population, EC^++^ media [human serum free endothelial medium (hESFM, Thermo Fisher, Waltham, MA, USA) supplemented with 1% bovine platelet-poor plasma-derived serum (PDS, Alfa Aesar, Ward Mill, MA, USA), 10ng/mL bFGF and 10 uM retinoic acid (RA)] was introduced for two days. Upon eight days of differentiation, the cells were purified on collagen IV/fibronectin surfaces (400 ug/mL collagen IV and 100 ug/mL fibronectin). bFGF and RA was removed from hESFM (EC^--^) for 24 h after purification for barrier maturation. Purified endothelial monolayers were employed for experiments on day 10.

### 2.2. Measurement of Barrier Function: TEER and Paracellular Permeability

Barrier integrity or monolayer tightness was assessed by measuring transendothelial electrical resistance (TEER) while using a Millicell ERS electrode (Millipore, Bedford, MA, USA). Paracellular permeability was assessed in monolayers by measuring the diffusion profile of 0.1 µCi of [^14^C] sucrose (PerkinElmer, Waltham, MA, USA). Radioactivities of samples were counted using a liquid scintillation counter (PerkinElmer, Waltham, MA, USA). The permeability coefficient was calculated while using following formula [34]:

$$PC=\frac{dQ}{dt}\times \frac{1}{A\times C_{0}}$$

where *dQ/dt* is rate of [^14^C] sucrose diffusion, *A* is area of insert, and *C*_0_ is the initial concentration of [^14^C] Sucrose added in donor compartment.

### 2.3. Measurement of Biphalin: LC-MS/MS Method

We measured the level of biphalin in cell lysate (uptake studies) and transport buffer (transport studies) while using our developed, highly sensitive LC-MS/MS method. Briefly, biphalin was extracted from the samples by the addition of acetonitrile (1:2) containing 10 ng/mL internal standard, [D-Ala^2^] Leucine Enkephalin (Sigma-Aldrich, St. Louis, MO, USA). After 1-min. vortexing, the mixture is centrifuged at 10,000 rpm for 10 min. After that the supernatant is mixed with an equivalent volume of LCMS water and then loaded into the insert vials ready for the analysis. The UHPLC-MS/MS system equipped with AB SCIEX QTRAP^®^ 5500 mass spectrometer (Foster City, CA, USA) attached to a Nexera ultra high performance liquid chromatography (UHPLC) system from Shimadzu Corporation (Columbia, MD, USA) was used. Data acquisition and quantification were performed by Analyst software 1.7.0.

Chromatographic separation was then performed with a Kinetex C18 column (50 × 2.1mm, 1.8 μm, Phenomenex, Torrance, CA, USA) while using mobile phases, 0.1% formic acid in water and 0.1% formic acid in acetonitrile. The gradients of acetonitrile (10% at time zero, 31% at 2.5 min., 90% at 2.6 min. up to 3.6, followed by 10% at 3.7 min. up to 4.5 min.) were used to elute biphalin at a flow rate of 0.4 mL/min. Column temperature was maintained at 45 °C. The retention time of biphalin was ∼1.8 min. while the retention time for the internal standard (IS) was ∼2.2 min. Multiple reaction monitoring was conducted in negative mode, and the transitions for biphalin and internal standard were 455.2 → 136.2 *m/z* and 570.3 → 120.3 *m/z*, respectively. The collision energy for biphalin and IS was 40 and 45 volts, respectively.

### 2.4. Biphalin Uptake Studies in iPSC-BMECs

Differentiated BMECs (iPSC-BMECs) are seeded in 12-well plates, incubated with either biphalin (10 µg/mL) (control) or pre-incubated with OATP1 substrate, estrone-3-sulfate (E3S) (10 µM) for 10 min., followed by co-incubation of E3S and 10 µg/mL biphalin in the 37 °C incubator for 20 min. The incubation time, 20 min. was selected to get detectable amount of biphalin in the cell extract. Next, the cells are washed two times with ice-cold phosphate buffered saline (PBS, pH 7.4). The cells were solubilized with 100 µL radioimmunoprecipitation assay (RIPA) Buffer (Thermo Fisher, Waltham, MA, USA) with protease and phosphatase inhibitor cocktail (Thermo Fisher, Waltham, MA, USA), and the amount of biphalin uptake was determined by using the above LCMS method. Protein content was calculated with a detergent-compatible bicinchoninic acid (BCA) assay (Pierce Chemical, Rockford, IL, USA). Biphalin uptake was normalized while using protein content and then presented as biphalin (ng) per µg of protein.

### 2.5. Biphalin Transcellular Transport Studies

Transport studies were performed to determine the role of OATP1 on the blood-to-brain transport of biphalin. Confluent iPSC-BMECs, (one million cells/insert) were seeded to the apical side of a 12-well transwell insert (area 1.12 cm^2^, polyester), which is pre-coated with 400 µg/mL collagen and 100 µg/mL fibronectin [35]. 10 µg/mL biphalin and an equivalent concentration of FITC-dextran (4kD) were added to the apical compartment [36]. For studies using an inhibitor and OATP1 substrate, E3S was pre-incubated for 30 min., followed by incubation with biphalin and E3S [11]. E3S was added to both the luminal and abluminal sides to inhibit the transporters on both sides. The results were expressed as a net permeability coefficient (PC) after subtracting the PC of FITC-dextran from the PC of biphalin. Apical-to-basolateral permeation coefficient was calculated while using following formula [34]:

$$PC=\frac{dQ}{dt}\times \frac{1}{A\times C_{0}}$$

where *dQ/dt* is rate of biphalin transport, *A* is area of insert, and *C*_0_ is the initial concentration of biphalin added in donor compartment.

### 2.6. Oxygen Glucose Deprivation (OGD) and Reperfusion

In vitro stroke conditions were simulated by exposing the cells to oxygen-glucose deprivation (OGD) conditions [37]. Briefly, iPSC-BMECs were rinsed twice with Dulbecco’s Modified Eagle medium Solution (DMEM). The cells were incubated with DMEM media (without glucose) plus 1% of PDS [33]. Hypoxia (1% oxygen) was induced by placing the cells in a hypoxic polymer glove box (Coy Laboratories, Grayslake, MI, USA), which was infused with 95% N_2_ and 5% CO_2_ at 37 °C. Cells were exposed to OGD condition for 1 h, 2 h, and 6 h, followed by reperfusion with OGD media with glucose and sodium pyruvate. For reperfusion studies, the cells were incubated in the CO_2_ incubator for pre-determined timepoints, 30 min., 1 h, 2 h, and 6 h.

### 2.7. Immunocytochemistry

The cells were seeded into four-well chamber slides (Lab-Tek II CC2 Chamber Slides), grown and fed for 24 h with EC^++^, followed by 24 h feeding with EC^--^. After experimental procedures, the cells were fixed with 4% paraformaldehyde (PFA), washed and permeabilized using 0.1% Triton X-100 for 5 min. The cells were blocked with 1% Bovine Serum Albumin (BSA) containing 2% goat serum (blocking solution) at room temperature for 1 h followed by incubation with the primary antibody in the same blocking solution (mouse monoclonal (mAb), OATP-A (1:100, Santa Cruz Biotechnology, Dallas, TX, USA) overnight at 4 °C [38]. Next day, the cells were washed three times with 0.1% BSA solution in PBS and were then stained with fluorescence-tagged corresponding secondary antibody at room temperature for 2 h followed by three times wash with PBS. Slides were mounted with 4,6-diamidino-2-phenylindole (DAPI) containing Prolong Gold antifade mounting media (Invitrogen). The slides were allowed to dry, protected from light. Slides were observed and images were obtained using a Nikon Eclipse Ti-E Epi-Fluorescence microscope. Mean fluorescence intensity (MFI) was calculated for each color channel while using Nikon industrial microscope software, NIS-elements AR (advanced research) software 4.13.05. The negative control was only incubated with secondary antibody.

### 2.8. Flow Cytometry

The cells were seeded into six-well cell culture plates, as described above. Single cell suspension was collected using accutase (Corning Inc., Corning, NY, USA), washed with phosphate buffered saline (PBS), and fixed with 4% PFA. The fixed cells were incubated with blocking solution containing with 10% goat serum (non-permeabilized) or 10% goat serum with 0.1% Tritox-X100 (permeabilized). The cells were collected by centrifugation, incubated with OATP-A antibody (1:50 dilution) overnight at 4 °C, followed by washing with PBS. Afterwards, the cells were incubated with rhodamine conjugated secondary antibody for 1 h at room temperature, washed with PBS and suspended in 1% BSA. The negative controls were incubated with isotype antibody. About 10,000 cells were analyzed while using the PE-A filter in a BD Bioscience FACSVERSE instrument. The MFI of negative controls was subtracted from the MFI of respective samples.

### 2.9. Data Analysis

All the data are presented as mean ± SD. Unpaired student’s *t*-test was used to compare two groups whereas to compare more than two groups one-way analysis of variance (ANOVA) followed by Tukey’s post hoc multiple comparison test was used (Prism, version 7.0; GraphPad Software Inc., San Diego, CA, USA). *P* values < 0.05 were considered to be statistically significant.

## 3. Results

### 3.1. Selection of OATP1 Expressing Brain Endothelial Cells

**Figure 1:**
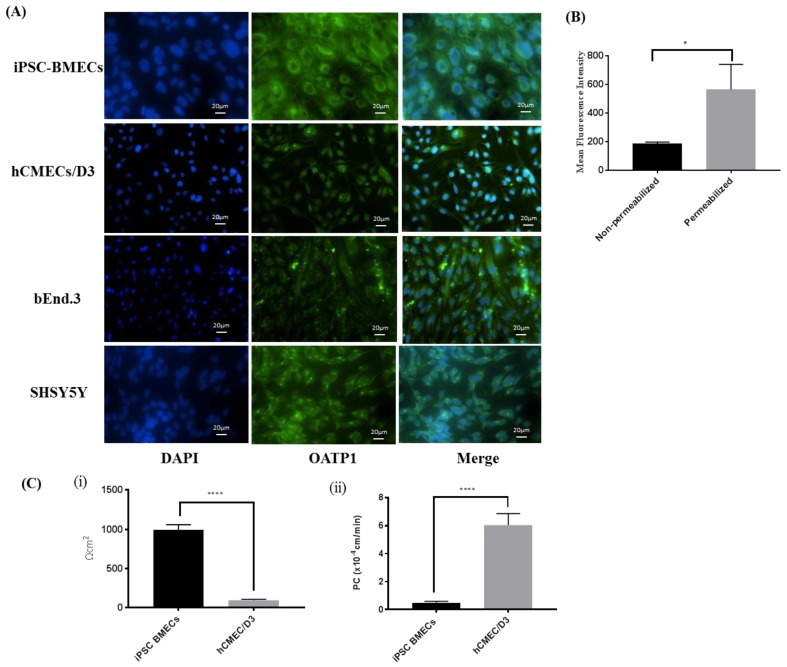
(**A**) Immunocytochemistry indicates positive expression of organic anion transporting polypeptide (OATP)1 in three different brain endothelial cells; two human (induced pluripotent stem cells differentiated brain microvascular endothelial cells (iPSC-BMECs) and hCMEC/D3) and one mouse (bEnd.3). SHSY5Y cells were used as positive control. The image clearly shows that there are both perinuclear (predominant) and membranous expression of OATP1 in iPSC-MBECs. Beside human cell lines, bEnd.3 also expresses this transporter, however comparatively lower than iPSC-BMEC and hCMEC/D3 cells. (**B**) The expression of membrane OATP1 was confirmed while using flow cytometry. Mean fluorescence intensity (MFI) was measured using permeabilized and non-permeabilized cells. Flow cytometry analysis data confirm the expression of OATP1 in iPSC-BMECs on membrane as well as in perinuclear region (**C**) Barrier function of the cells was measured using transendothelial electrical resistance (TEER) (**C-i**) and [^14^C] sucrose permeability (**C-ii**) across cells monolayer. The iPSC-BMECs exhibit restrictive barrier properties as compared to hCMEC/D3 demonstrated by ten times higher TEER and ten times lower paracellular permeability. Data represented as Mean ± SD (*n* = 5). * *p* < 0.05, **** *p* < 0.0001.

Two human originated brain endothelial cells, hCMEC/D3 and iPSC-BMECs, and one mouse originated, bEnd.3 cells, were used to measure the comparative expression of OATP1 by using immunocytochemistry. SHSY5Y neuroblastoma cells were used a positive control, as per supplier’s protocol. The results of the study (Figure 1A) showed that all three cells expressed OATP1, but human originated cells expressed comparatively higher OATP1. Moreover, we also observed that iPSC-BMECs expressed higher OATP1 compared to hCMEC/D3. We observed perinuclear as well as membrane expression of OATP1 in iPSC-BMECs. Non-permeabilized and permeabilized cells were stained with OATP antibody followed by fluorescence tagged secondary antibody and analyzed using flow cytometry to measure the membrane portion of OATP1. We observed higher mean fluorescence intensity when cells were permeabilized while using Triton-X100 as compared to non-permeabilized cells (Figure 1B). It was also found that nearly 25% OATP1 expressed on membrane of the cells, which could contribute in the uptake and transport of substrates across the BBB.

The barrier tightness between two human cells, hCMEC/D3 and iPSC-BMEcs, was measured by using two well reported techniques, i.e., TEER and [^14^C] paracellular permeability (Figure 1(C-i)) to further optimize the cells for uptake and transport studies. Results of the study showed that iPSC-BMECs demonstrated significantly (*p* < 0.0001) higher TEER value when compared to hCMEC/D3 (1000 ± 100 Ω.cm^2^ vs 100 ± 20 Ω.cm^2^).

Beside TEER, [^14^C] sucrose permeability coefficient (PC), which indicates paracellular leakiness across the iPSC-BMECs monolayer, was found to be 0.45 ± 0.13 × 10^−4^ cm/min. as compared to 6 ± 0.8 × 10^−4^ cm/min. in hCMEC/D3 cells (Figure 1(C-ii)). These studies demonstrate that iPSC-BMECs possess significantly higher barrier tightness than hCMEC/D3 cells. iPSC-BMECs were chosen for further studies based on the results of these studies.

### 3.2. OATP1 Contributes to Biphalin Uptake and Transport

**Figure 2:**
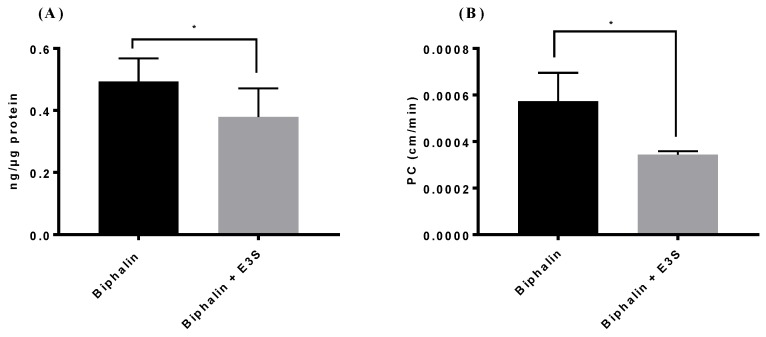
Uptake (**A**) and transport (**B**) studies using iPSC-MBECs demonstrated that biphalin uptake in the endothelial cells (ECs) and transport across the blood-brain barrier (BBB) decreased in the presence of OATP1 inhibitors. (**A**) For uptake studies, the cells were pre-incubated with estrone-3-sulfate (E3S) for 10 min. before incubating with mixture of biphalin (10 µM) and inhibitor (E3S, 10 µM) for 20 min. (**B**) For transport studies, monolayer cells were pre-treated 30 min. with OATP1 inhibitor. A mixture of biphalin and inhibitor was added to the apical side and samples were collected from basolateral side (A to B transport). FITC-dextran 4kD was used to correct the paracellular permeability. Presented permeability coefficient (PC) is the net difference of PC of biphalin and FITC-dextran. Data represented as Mean ± SD (*n* = 4). * *p* < 0.05.

We measured the uptake (Figure 2A) and transport (Figure 2B) of biphalin in iPSC-MBECs in normal conditions to determine the role of OATP1 in the transport of biphalin across the BBB during ischemic stroke. The results of the studies showed that after incubation of biphalin for 20 min. with iPSC-BMECs, the uptake values were 0.49 ± 0.05 ng of biphalin per 1 ug of protein. However, when the cells were pre-treated with E3S, an OATP1 substrate, for 10 min. before biphalin incubation, the uptake of biphalin significantly reduced (*p* < 0.05) to 0.37 ± 0.05 ng/ug protein.

For transport studies, iPSC-BMECs monolayers that were grown on collagen-fibronectin coated polyester transwell inserts were used as an in vitro BBB model. A permeability coefficient (PC) of biphalin (apical to basolateral) was calculated as the net difference between biphalin permeability and FITC-dextran (4 kD) permeability. The results of the study showed that biphalin crossed the cell monolayer with a PC value of (0.00057 ± 0.0001) cm/min. Similar to uptake studies, 30 min. prior treatment with E3S resulted in the significant reduction of biphalin PC (0.00034 ± 0.00002 cm/min. with E3S vs 0.00057 ± 0.00001 cm/min. without E3S). The results of these studies suggest that biphalin uses OATP1 to transport across the BBB. We also measured the transport of biphalin from basolateral to apical side (B to A) to determine the role of P-gp, or other efflux mechanisms, and it was found to be negligible (data not shown).

### 3.3. Effect of OGD-Reperfusion on Biphalin Uptake

We measured the effect of oxygen-glucose deprivation (OGD)-reperfusion (to model stroke injury) on biphalin uptake in iPSC-BMECs after confirming the role of OATP1 in the uptake and transport of biphalin across the normoxic BBB.

**Figure 3:**
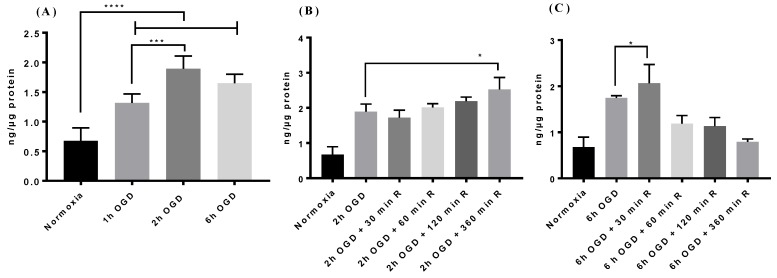
The effect of oxygen-glucose deprivation (OGD) exposure on biphalin uptake. (**A**) Biphalin uptake significantly increased after different times of OGD exposure; maximum after 2 h OGD (**B**) Biphalin uptake while reperfusion after 2 h OGD tend to increase time dependently, and it becomes significant at 2 h OGD and 6 h R. (**C**) Biphalin uptake increased after 30 min. reperfusion after 6 h OGD; however, it decreased during extended reperfusion times until reaching the basal normoxic level at 6 h reperfusion. Data represented as Mean ± SD (*n* = 4). * *p* < 0.05, *** *p* < 0.001, **** *p* < 0.0001.

We observed that biphalin uptake significantly increased after 1 h (1.9-fold), 2 h (2.8 fold), and 6 h (2.4 fold) of OGD exposure as compared to the uptake in normoxic conditions (*p* < 0.0001) (Figure 3A). Interestingly, 2 h OGD exposure resulted in significantly higher uptake when compared to 1 h OGD (1.89 ± 0.015 ng/ug vs 1.31 ± 0.2 ng/ug) and no significant difference was observed between 2 h and 6 h OGD exposure (1.89 ± 0.015 ng/ug vs 1.64 ± 0.15 ng/ug).

Based on the results of uptake studies after OGD exposure, we selected 2 and 6 h OGD time points to determine the effect of different reperfusion times on the biphalin uptake. We observed that, after 2 h OGD exposure, biphalin cellular uptake was not significantly increased until 6 h reperfusion (2.5 ± 0.2 ng/ug at 2 h OGD + 6 h reperfusion vs 1.89 ± 0.015 ng/ug after 2 h OGD) (Figure 3B). However, after 6 h OGD exposure, biphalin uptake significantly increased (*p* < 0.05) at earlier time points (2 ± 0.25 ng/ug at 30 min.) and reduced slightly up to 2 h during reperfusion when compared to 6 h OGD, but still significantly higher (*p* < 0.01) than normoxic conditions (Figure 3C). After 6 h of reperfusion, biphalin uptake in the cells decreased to the basal normoxic level.

### 3.4. OATP1 Expression Increased During OGD-Reperfusion

**Figure 4:**
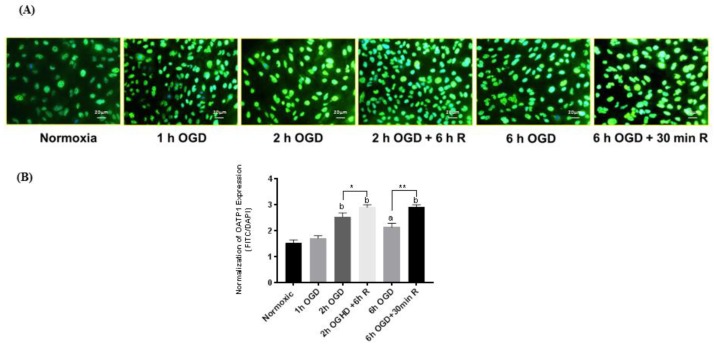
(**A**) Immunofluorescence images of iPSC-BMECs after staining with OATP1 antibody at different OGD)-reperfusion time points. (**B**) Quantitative measurement of the changes in OATP1 expression after normalizing mean fluorescence intensity of OATP1 with that of 4,6-diamidino-2-phenylindole (DAPI). The expression of OATP1 in iPSC-BMECs increased after 2 and 6 h OGD exposure which further increased during reperfusion. Data represented as Mean ± SD (*n* = 4). * *p* < 0.05, ** *p* < 0.01. *a* < 0.001, *b* < 0.0001 vs normoxic.

After finding that biphalin uptake increases after different OGD exposures and also at selective reperfusion times after OGD, we wanted to determine the effect of OGD-reperfusion on the expression profile of OATP1. After exposing cells for predetermined OGD and OGD-reperfusion time periods, cells were probed for OATP1 expression using an immunofluorescence technique (Figure 4A). Interestingly, we found that OATP1 expression increased correspondingly at the same time points that we observed significant increases in biphalin uptake, i.e., 2 h OGD + 6 h reperfusion and 6 h OGD + 30 min. reperfusion. After normalizing MFI of OATP1 with the corresponding DAPI MFI, we found that the expression of OATP1 slightly but not significant increased at 1 h OGD, however as expected, significantly higher expression was observed after 2 h (*p* < 0.0001), 2 h OGD + 6 h reperfusion (*p* < 0.0001), 6 h OGD (*p* < 0.001), and 6 h OGD + 30 min. reperfusion (*p* < 0.0001) when compared to normoxic control (Figure 4B). It was also observed that compared to OGD exposure, OATP1 expression further increased during reperfusion when 2 h OGD + 6 h reperfusion was significantly higher than 2 h OGD (*p* < 0.05) and 6 h OGD + 30 min. reperfusion was significantly higher than 6 h OGD (*p* < 0.01).

### 3.5. OATP1 Contributes in the Uptake and Transport of Biphalin across the BBB

**Figure 5:**
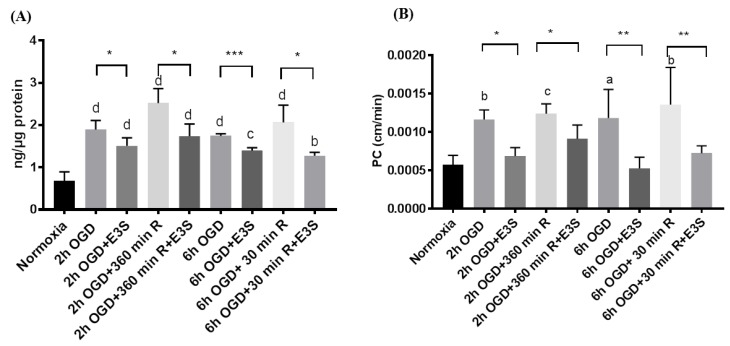
Biphalin uptake (**A**) into iPSC-BMECs and transport (**B**) across iPSC-BMECs monolayer decreased significantly when the cells were pre-incubated with competitive OATP1 substrate, E3S. Data represented as Mean ± SD (*n* = 3). * *p* < 0.05, ** *p* < 0.01, *** *p* < 0.001.

Biphalin uptake into iPSC-BMECs and transport across iPSC-BMEC monolayers was measured in the presence and absence of OATP1 substrate, E3S. We selected four time points for uptake (Figure 5A) and transport studies (Figure 5B) based on the results of uptake studies and immunofluorescence assay, which were 2 and 6 h OGD exposure and 6 h reperfusion after 2 h OGD and 30 min. reperfusion after 6 h OGD. The cells were incubated with E3S (10 min. for uptake studies, whereas 30 min. for transport studies) before incubating them with biphalin. We found that biphalin uptake significantly decreased (*p* < 0.05) in the presence of E3S in comparison to the corresponding time points without E3S pre-incubation (Figure 5A). Similar results were observed in the transport studies (Figure 5B), where biphalin transport across the monolayer increased after 2 and 6 h OGD exposure, but significantly decreased in the presence of OATP1 substrate E3S.

## 4. Discussion

The neuroprotective role of opioid receptor agonists in stroke has been well reported earlier by our lab and other researchers [4,5,6,7,8,39,40,41]. It has also been documented that non-selective ORs agonists improve the stroke outcomes better than selective agonists [7]. Biphalin is an example of non-selective, ultra-potent, peptide based OR agonist that has higher affinity to MOR and DOR and less affinity to KOR [42,43]. The original rationale for the synthesis of biphalin was the higher probability of interaction of the molecules with two active sites of the receptor, and also of unique interaction with proteolytic systems [44]. Biphalin has been well studied for its analgesic activity during inflammatory pain and has shown better activity than morphine when intracerebrally administered [45]. These unique properties of biphalin, high receptor affinity to MOR, DOR, and KOR and potent antinociceptive activity, are the results of the rigidity of the hydrazide bond between two active chains [46]. It has been proposed for the treatment of abdominal pain that is associated with inflammatory bowel disease [47] and has also been proposed to be better than morphine in the management of cancer associated pain [43]. With this pharmacological profile, biphalin remains one of the best preclinical peptides based opioid analgesics [42,48]. Most importantly, biphalin exerts less dependence and tolerance than the classical opioid receptor antagonist morphine [42,49,50]. This could be attributed to its affinity to KOR, which results in combating the MOR and/or DOR agonist-mediated adverse effects, like psychological and physical dependency, and tolerance [51].

It was reported that biphalin works better than other selective opioid agonists in decreasing edema formation and infarct size ratio in mice middle cerebral artery occlusion (MCAO) model when administered 10 min. after reperfusion [6]. In addition, biphalin demonstrated equivalent efficacy to the best lead of the non-selective fentanyl derivatives, LYS739 [8]. However, no study has fully characterized the transporters that are involved in the transport of biphalin across the BBB both under normal and stroke conditions. The objective of present study was to characterize the role of OATP1 in the transport mechanism of biphalin during ischemic stroke in an in vitro model while using human originated cells.

We screened brain endothelial cells that express OATP1 transporters and also possess restrictive permeability properties to choose an in vitro cell model. Two human cell lines; hCMEC/D3 and iPSC derived BMECs, expressed OATP1 transporters at a higher level than bEnd.3 cells (Figure 1A). Further, iPSC-BMECs exhibited higher barrier properties in terms of higher TEER and lower PC for [^14^C] sucrose as compared to other human originated OATP1 expressing cells, hCMEC/D3 (Figure 1B). Hence, for further studies, iPSC-BMECs were used for our in vitro cell model of the BBB. The iPSCs derived BMECs also provide strong translational potential due to their human origin cells. We wanted to study the effect of OGD and OGD/reperfusion on the expression profile of OATP1 in human cells with respect to biphalin transport across the ischemic BBB. Before that, we wanted to confirm if OATP1 contributes to the uptake and transport of biphalin across the BBB in normal conditions. It was found that the pre-incubation of an OATP substrate (E3S) reduced the uptake of biphalin in iPSC-BMECs (*p* < 0.05) (Figure 2A). Similar results were found in an in vitro transwell model, where the permeability of biphalin was reduced in the presence of the OATP1 substrate (*p* < 0.05) (Figure 2B). Interestingly, E3S, a selective OATP1A2 substrate, only reduced a part of biphalin uptake and transport (approximately 23 and 40%, respectively). Thus, it could be suggested that the OATP1 transporters play a partial role in the transport of biphalin in normal conditions. It was also reported earlier that biphalin uses a carrier-mediated transport process to cross the BBB [36,52]. We also studied the effect of OGD and OGD/R on the uptake of biphalin by iPSC-BMECs. We selected different OGD time-points, 1, 2, and 6 h, to measure the uptake of biphalin in OGD- reperfusion conditions. As expected, we found that biphalin uptake increased after OGD exposures (1.9, 2.8, and 2.4 times higher than normoxia after 1, 2, and 6 h, respectively); significantly higher after 2 h as compared to 1 h (*p* < 0.001) and it remained higher up to 6 h exposure. Based on the results of OGD exposure, we selected 2 and 6 h time-points for re-oxygenation. Interestingly, we found that, after 2 h of OGD, biphalin uptake increased with time during reperfusion and reached significance after 6 h reperfusion (*p* < 0.05), whereas after 6 h of OGD, biphalin uptake increased during 30 min. reperfusion and decreased when re-oxygenation time extended to 6 h (Figure 3B,C). We measured the expression of OATP1 in iPSC-BMECs at selected timepoints where biphalin uptake was found to be high, i.e., 2 h OGD, 6 h OGD, 2 h OGD plus 6 h reperfusion, and 6 h OGD plus 30 min. reperfusion to confirm the role of OATP1 in the increased biphalin uptake during OGD- reperfusion. The results of our study demonstrated that OATP1 expression significantly increased when the cells were exposed to 2 h in OGD conditions (*p* < 0.001), which slightly reduced after 6 h of OGD, but still significantly higher than normoxic condition (*p* < 0.01) (Figure 4). We also found that OATP1 expression increased during reperfusion timepoints that are similar to uptake profile, i.e., 2 h OGD plus 6 h reperfusion, and 6 h OGD plus 30 min. reperfusion. Results of these studies partially confirmed the role of OATP1 transporters in the uptake of biphalin by brain endothelial cells. Thompson et al. have also reported a similar pattern [11], where they reported increased expression of oatp1a4 in rats during 30 min. reoxygenation after 1 h of hypoxia, which further reduced during longer reoxygenation. It has also been reported that atorvastatin and taurocholate brain uptake increased when oatp1a4 expression increased in different disease models and reduced in the presence of competitive oatp1a4 substrates, estrone-3-sulfate, and fexofenadine [10,11]. However, no study has reported the expression profile and the effect of disease models in human originated cells. Additionally, no study has measured the transport mechanism of biphalin across the BBB during OGD/reperfusion conditions to simulate stroke conditions.

We evaluated the role of OATP1 on the uptake and transport of biphalin across the in vitro BBB during an in vitro model of stroke (OGD-reperfusion) after confirming the expression of OATP1 and the effect of OGD and OGD/reperfusion on its expression. Based on the expression profile during OGD and OGD-reperfusion, we selected different OGD time-points to measure the uptake of biphalin in OGD-reoxygenation conditions in the presence of OATP1 substrate, E3S. We found that pre-incubation significantly reduced the uptake of biphalin at all of the selected timepoints (average reduction, 26.59 ± 8.01%) (Figure 5A). Hence, it could be concluded that OATP1 contributes to the increased uptake of biphalin during OGD and reperfusion.

As reported earlier, OATP1A2 transporters are expressed on the luminal membrane of the BBB [53] and play a crucial role in the transport of various therapeutic molecules including analgesic opioid peptides [19], lipid lowering agents [54], as well as cancer medications [38,55,56]. We performed the apical-to-basolateral permeability studies using an in vitro transwell model after seeding iPSC-BMECs on collagen-fibronectin coated polyester inserts. To study the effect of OATP1 transporters, the cells were pre-treated with OATP1 substrate, E3S for 30 min. As expected, transport of biphalin across the BBB increased after 2 and 6 h of OGD exposure and also during reperfusion timepoints (2 h OGD plus 6 h reperfusion and 6 h OGD plus 30 min. reperfusion) also suggesting the role of OATP1 in the transport of biphalin. This was further confirmed when biphalin transport decreased significantly in the presence of OATP1 substrate, E3S (average reduction, 37.96 ± 12.51%) (Figure 5B).

As discussed earlier, biphalin has been shown to be an effective neuroprotective agent in stroke and, as such, needs to be transported across the BBB. In this study we observed that after shorter durations of OGD exposure, 2 h, the expression of OATP1 increased further up to 6 h during reperfusion. However, when the cells were exposed for longer duration of OGD, 6 h, the expression of OATP1 increased only initially at 30 min. during reperfusion and subsequently reduced to normal level after 6 h. Hence, the findings of our study not only will help to understand the mechanism of increased biphalin uptake/transport across the BBB during ischemic stroke, but also contribute in understanding the time-course profile of biphalin administration for optimal delivery to the ischemic brain. As we know, tissue plasminogen activator (t-PA) is the only US Food and Drug Administration (FDA) approved drug for acute stroke treatment, and its clinical use is limited by its narrow therapeutic time window (~4.5 h) and propensity to cause hemorrhagic transformations (HT) after cerebral ischemia [57]. Mechanical thrombectomy, which is a method to open large vessels, has also shown a beneficial effect in ischemic stroke [58]. In addition to targeted reperfusion strategies, additional neuroprotective strategies have been a promising area in stroke research, with the goal to enhance neuronal survival and improve stroke outcome both with and without reperfusion [59]. Based on the findings of our studies, it could be suggested that, after shorter periods of occlusion, i.e., in t-PA therapeutic window, biphalin could be administered after longer periods of reperfusion and provide possible cerebroprotection. Additionally, after an extended duration of occlusion, possibly beyond the t-PA therapeutic window, biphalin could be administered during the re-vascularization process or after short periods of reperfusion time after revascularization. Further in vivo studies are warranted to explore these therapeutic opportunities in stroke.

Future studies are warranted to determine the effect of in vivo stroke models (ischemia–reperfusion) on the expression profile of OATP1 since we found a significant role of OATP1 in the transport of biphalin across BBB during ischemic stroke in an in vitro model. Although it has already been reported by the Ronaldson Lab that TGF-β/ALK-5 signaling plays a key role in the regulation of oatp1a4 expression in rat brain capillary endothelial cells in pathological stress like inflammatory pain and hypoxia/reoxygention [10,11], it is also critical to consider the signaling mechanism behind the increased expression of OATP1 during ischemia reperfusion. As the BBB is a complex, dynamic, and adaptable barrier in which communication between endothelial cells and other constituent cells, like astrocytes, pericytes, and neurons affects the barrier integrity and the expression of transporters in physiological changes and during pathological conditions [60,61]. We also expect that neurovascular cells, particularly astrocytes and pericytes, affect the expression of OATP1 during ischemic stroke. Hence, transport studies using a co-culture model of iPSC-BMECs and iPSCs differentiated glial cells would be interesting to determine the impact of other neurovascular cells on the expression of OATP1 and on the transport of biphalin across the BBB during ischemic stroke. Furthermore, it would be interesting to investigate the in vivo distribution of biphalin between the ipsilateral and contralateral hemispheres during ischemia-reperfusion. The results of the present study provide an opportunity to optimally design a preclinical study to test the neuroprotective potential of biphalin or other OR agonists, as a standalone stroke treatment or in combination with t-PA/thrombectomy procedures. Additionally, it will be important to test whether other structurally related molecules utilize OATP1 as a possible mechanism to access ischemic penumbral tissue and aid in stroke recovery.
